# Mapping direct and indirect MarA/SoxS/Rob/RamA regulons in *Salmonella* Typhimurium reveals repression of *csgD* and biofilm formation

**DOI:** 10.1099/mic.0.001330

**Published:** 2023-05-19

**Authors:** Alistair D. Middlemiss, James R. J. Haycocks, Anne M. Stringer, Laura J. V. Piddock, Joseph T. Wade, David C. Grainger

**Affiliations:** ^1^​ School of Biosciences, University of Birmingham, Edgbaston, Birmingham B15 2TT, UK; ^2^​ Wadsworth Center, New York State Department of Health, Albany, New York, USA; ^3^​ Institute of Microbiology and Infection, University of Birmingham, Edgbaston, Birmingham, B15 2TT, UK; ^4^​ Department of Biomedical Sciences, School of Public Health, University at Albany, Albany, New York, USA; ^†^​ Present address: Global Antibiotic R&D Partnership, 15 Chemin Camille-Vidart, 1202 Geneva, Switzerland

**Keywords:** antibiotic resistance, transcription, genomics, gene regulation, AraC family

## Abstract

The closely related transcription factors MarA, SoxS, Rob and RamA control overlapping stress responses in many enteric bacteria. Furthermore, constitutive expression of such regulators is linked to clinical antibiotic resistance. In this work we have mapped the binding of MarA, SoxS, Rob and RamA across the *Salmonella* Typhimurium genome. In parallel, we have monitored changes in transcription start site use resulting from expression of the regulators. Together, these data allow direct and indirect gene regulatory effects to be disentangled. Promoter architecture across the regulon can also be deduced. At a phylogenetic scale, around one third of regulatory targets are conserved in most organisms encoding MarA, SoxS, Rob or RamA. We focused our attention on the control of *csgD*, which encodes a transcriptional activator responsible for stimulating production of curli fibres during biofilm formation. We show that expression of *csgD* is particularly sensitive to SoxS that binds upstream to repress transcription. This differs to the situation in *Escherichia coli *, where MarA regulates *csgD* indirectly.

## Introduction

In *Escherichia coli *, the multiple antibiotic resistance (*mar*) locus was identified in a screen for mutations conferring resistance to tetracycline [1]. Subsequent testing revealed cross-resistance to quinolones and β-lactams [1]. The locus encompasses the *marRAB* operon encoding MarR (a transcriptional auto-repressor), MarA (a global regulator) and MarB (a poorly understood membrane-associated protein) [2]. Mutations giving rise to drug resistance hinder autorepression by MarR and, as a result, MarA is overexpressed [2, 3]. Subsequent changes in global transcription give rise to the *mar* phenotype [4]. In wild-type cells, the ability of MarR to autoregulate *marRAB* is influenced by phenolic compounds and so expression of MarA occurs in response to stress. Following the discovery of the *mar* locus, other laboratories identified analogous stress response systems elsewhere in the genome. In *E. coli *, the *soxRS* locus consists of divergent genes with a shared regulatory region. In this scenario, repression of *soxS* by SoxR is relieved by superoxide stress. Expression of SoxS results and the regulator alters gene expression accordingly. Crucially, SoxS and MarA share 42 % sequence identity, bind the same target DNA sequence, and so have overlapping regulatory effects [4, 5]. Hence, overexpression of SoxS can also give rise to clinically relevant antibiotic resistance [6]. Both MarA and SoxS belong to the AraC family of transcription factors [7]. Such proteins are distinguished by the presence of a dual helix-turn-helix (HTH) motif DNA binding domain [7]. Whilst most AraC family proteins have additional signal-sensing domains, MarA and SoxS do not. Instead, the cognate proteins MarR and SoxR sense environmental signals and regulate the levels of MarA and SoxS, respectively [8, 9].

The right of origin binding protein (Rob) was first identified as a factor associated with the chromosomal replication origin in *E. coli* [10]. Further examination revealed an N-terminal dual HTH domain 51 and 55 % identical to MarA and SoxS, respectively [11]. Hence, Rob preferentially binds to the same DNA sequence as MarA and SoxS. However, Rob binds DNA more promiscuously and with a much higher affinity than either MarA or SoxS [4, 12–14]. The reasons for this are unclear but an early structural study showed one of the two Rob HTH motifs sat atop the minor groove, rather than within the major groove as observed for MarA and SoxS [13]. The physiological relevance is unknown as more recent studies show association of both HTH motifs with adjacent sections of the major groove [15]. Unlike MarA and SoxS, Rob is not dependent on a regulator acting upstream to perceive environmental stress. Instead, the Rob C-terminal domain drives aggregation of the protein in standard growth conditions. These clusters are dispersed by interactions between the C-terminal domain and dipyridyl, releasing active Rob [16]. Rob controls a regulon overlapping those of MarA and SoxS.

Homologues of *marA*, *soxS* and *rob* are encoded by many enteric bacteria. In addition, some organisms encode a closely related paralogue, RamA. RamA was first described in *Klebsiella pneumoniae* where it elicits a *mar*-like phenotype. *K. pneumoniae* RamA shares 42 % sequence identity with *E. coli* MarA [17]. Whilst not found in *E. coli* and *Shigella* spp., RamA is encoded by other enterobacteriaceae including some *Salmonella* spp. [18]. Thus, there are many examples of clinical antibiotic resistance associated with altered levels of RamA [19–23]. Deciphering the complete set of genes regulated by MarA, SoxS, Rob and, if encoded RamA, has been complicated by pleiotropy, redundancy between the factors, and degeneracy of the DNA consensus sequence [24]. In this study, we have focused on the MarA, SoxS, Rob and RamA regulons of *S*. Typhimurium SL1344. Using chromatin immunoprecipitation, coupled with Illumina sequencing (ChIP-seq), we have mapped genome-wide DNA binding by MarA, SoxS, Rob and RamA. Combined with genome-scale analysis of RNA transcript 5′ ends, this allowed mapping of direct and indirect regulons for each factor. Promoter architecture of the regulated genes was also determined. We show substantial overlap between the direct and indirect regulatory targets of the four proteins. Phylogenetically, around one third of the direct *S*. Typhimurium regulon is conserved in nearly all other Enterobacteriaceae examined. Investigation of direct target genes in *S*. Typhimurium identified *csgD*, which encodes a transcription factor required for biofilm formation. We show that expression of *csgD*, and associated biofilm formation, is particularly sensitive to direct repression by SoxS.

## Methods

### Strains, plasmids and oligonucleotides

Descriptions of strains, plasmids and oligonucleotides are provided in Table S1, available in the online version of this article. Standard microbiological techniques were used throughout. Growth conditions are provided in the relevant sections below and cultures were incubated at 37 °C unless stated otherwise.

### β-galactosidase assays

Measurements of promoter activity *in vivo* were done according to the Miller method [25]. Briefly, cells were grown until mid-log phase in LB medium supplemented with appropriate antibiotics. Following lysis, levels of lysate β-galatosidase activity were determined and normalized between samples according to the final OD_650_ of the culture. Values shown are the mean of three biological replicates and error bars show standard deviation.

### Chromatin immunoprecipitation and DNA sequencing

All ChIP-seq experiments follow the procedure described previously and were done in duplicate [4]. An overnight culture of *S*. Typhimurium SL1344, carrying the appropriate plasmid, was used to inoculate 40 ml of fresh LB medium. The resulting culture was grown to mid-log phase. Crosslinking was initiated with 1% (*v/v*) formaldehyde and allowed to proceed for 20 mins before quenching with 10 ml 2.5 M glycine. Cells were collected by centrifugation at 1600 ***g*** for 5 mins and sequentially washed with 25 ml, and then 1.5 ml, of 1×TBS. Cells were pelleted by centrifugation after each wash. Next, the cell pellet was re-suspended in 1 ml IP buffer [50 mM Hepes-KOH pH7, 1 mM EDTA, 1 % (*w/v*) Triton X-100, 0.1 % (*w/v*) sodium deoxycholate, 0.1 % (*w/v*) SDS] with 150 mM NaCl and either 2 or 4 mg ml^−1^ lysozyme. Following incubation at 37 °C for 30 min, the suspension was briefly chilled on ice before sonication with a Bioruptor Plus (Diagenode) for 30 cycles of 30 s on 30 s off at 4 °C. Cell debris was removed by centrifugation at 21 000 ***g*** for 5 mins and the supernatant divided into four microfuge tubes and diluted with 800 µl of IP buffer with 150 mM NaCl. One aliquot of the lysate was used per immunoprecipitation. Protein A sepharose beads were washed, then resuspended as a 50 % (*v/v*) slurry, using 1×TBS. Blunt pipette tips were used in these and all subsequent steps to avoid damaging the beads. For precipitations, 25 µl of Protein A beads, and 2 µl of the appropriate antibody, were added to the aliquot of cell lysate. The cocktails were incubated at room temperature, with constant mixing by inversion, for 90 min.

After immunoprecipitation, Protein A beads were collected by centrifugation at 1600 ***g*** for 1 min before resuspending in 700 µl fresh IP buffer with 150 mM NaCl. The mixtures were transferred to Spin-X columns and mixed for 3 min at room temperature. The buffer was removed by centrifuged at 1600 ***g*** for 1 min and discarded. After equivalent wash steps with IP buffer with 150 NaCl, and then with 10 mM Tris-HCl pH 7.5 (the latter done twice) DNA fragments were blunted using a quick blunting kit (NEB). Note that these reactions were set up in the Spin-X columns and mixed at room temperature for 30 min without inversion. The beads were then washed twice each with IP buffer containing 150 mM NaCl and 10 mM Tris-HCl pH 8. The next enzymatic step added an ‘A tail’ to each DNA fragment using the Klenow fragment (3′>5′ exo-). The reactions were incubated at 37 °C for 30 min with rotation but not inversion. Further washes (twice each) were done using IP buffer having 150 mM NaCl and 10 mM Tris-HCl pH 7.5. The final enzymatic step used ligation to add NEXTflex barcoded adaptors (BioOscientific) to the DNA fragments. Beads were then washed twice in IP buffer with 150 mM NaCl. Single wash steps were then done with IP buffer containing 500 mM NaCl, ChIP wash buffer [10 mM Tris-HCl pH 8.0, 250 mM LiCl, 1 mM EDTA, 0.5 % (w/v) Nonidet-P40, 0.5 % (w/v) Sodium Deoxycholate] and TE. DNA was eluted by transferring the Spin-X column basket to a fresh dolphin-nosed tube and incubated at 65 °C for 10 min in 100 µl ChIP elution buffer [50 mM Tris-HCl pH 7.5, 10 mM ETDA, 1 % (*w/v*) SDS]. Following incubation, reactions were transferred to a centrifuge and the eluate collected. Samples were then de-crosslinked by boiling for 10 min.

Prior to library amplification, DNA fragments were subjected to a 1.1×volume Agencourt AMPure XP bead clean up and eluted in 13 µl ddH2O. Next, 2 µl of the library was used in a qPCR reaction to determine the number of amplification cycles needed to maximized library amplification but minimised both NEXTflex barcode adapter and PCR primer dimers. Amplified libraries were diluted to 200 µl with ddH2O and subjected to a 0.7×AMPure XP bead clean up before imaging on an Agilent TapeStation 2200 (Agilent). Library concentration was quantified using an NEBNext Library Quant Kit (NEB) then adjusted to between 0.5 and 2 nM. Samples were then pooled and sequenced using an Illumina MiSeq. Sequence reads are accessible in ArrayExpress (accession number E-MTAB-12627).

### Bioinformatic analysis of ChIP-seq data

Bioinformatic analysis of ChIP-seq data was done as described previously [4]. Raw FASTQ files were converted to FASTQ Sanger format using FASTQGroomer and aligned to the *S. Typhimurium* SL1344 chromosome (NC_016810.1) or plasmids pCol1B9 (NC_017718.1), pRSF1010 (NC_017719.1) and pSLT (NC_017720.1) using Bowtie 2 for Illumina [26]. The resulting files were then converted to BAM format using SAM-to-BAM before determining the coverage per base using multiBamSummary. Further analysis was done using R. Each dataset was normalized to have the same average read depth and mean coverage per base calculated. The coverage values generated from mock immunoprecipitations were subtracted from the MarA, SoxS, Rob and RamA immunoprecipitation samples and signals at rRNA and tRNA genes removed. The resulting coverage plots were visualized using Artemis or DNA plotter [27, 28]. To select peaks, we identified chromosomal regions where the signal was at least 2.5 times the average read depth across 140 or more consecutive base pairs. MEME was used to identify binding sequences in 201 bp DNA sequences centred on each peak. To assess the phylogenetic conservation of binding sites the 201 bp sequences were submitted to blastn and used to search the genomes of the strains shown in Fig. 2(a) as described previously [4].

### Cappable-seq

This method exploits the ability of vaccinia capping enzyme to specifically modify triphosphorylated RNA 5′ ends with biotinylated GTP. This allows primary unprocessed transcripts (as opposed to processed RNAs, with monophosphorylated 5′ ends) to be isolated using streptavidin beads [29]. Cappable-seq was done by Vertis Biotechnologie AG on 5 µg of RNA extracted from *S*. Typhimurium SL1344 carrying pAMNF or pAMNM derivatives encoding epitope tagged derivatives of MarA, SoxS or RamA. Cells were grown to mid-log phase in LB medium. RNA extraction was done using the SV Total RNA Isolation System (Promega). Sequence reads are accessible in ArrayExpress (accession number E-MTAB-12628). As a control, we used cappable-seq data for *S*. Typhimurium SL1344 carrying empty pAMNF (E-MTAB-12506) [30].

### Bioinformatic analysis of cappable-seq data

Sequencing reads were mapped to the *S*. Typhimurium SL1344 reference genome (FQ312003.1, NC_017718.1, NC_017719.1 and NC_017720.1) using Bowtie2 and SAMtools (version 1.3.1). Transcription start sites were identified using the software of Ettwiller *et al*. [29]. Briefly, bam2firstbasegtf.pl was used to generate the .gtf files and relative read scores (RRSs). The latter represents the number of reads normalized to the total number of reads in the sample. The results are then filtered based on a cut-off value of 1.5 (equivalent to 20 reads or more). Cluster_tss.pl was used select the primary TSS, with the highest RRS, from small clusters of adjacent TSSs for the same promoter. To quantify changes in the signal at each TSS, resulting from expression of MarA, SoxS or RamA, we used EdgeR [31]. Volcano plots were generated using ggplot2 [32]. DNA sequence logos were generated using Weblogo [33]. When categorizing promoters, those with MarA, SoxS or RamA binding motifs in the reverse orientation, located between 53 and 73 bp upstream of the TSS, were defined as class I. Binding motifs in the forward orientation, between 30 and 42 bp upstream of the TSS, designated promoters as class II.

### Proteins

Genes encoding *S*. Typhimurium MarA, SoxS, Rob or RamA were cloned in pET28a and His_6_ tagged variants overexpressed in *E. coli* T7 Express cells. Purification was as described by Kettles *et al*. [34]. Purified proteins were concentrated to 1 mg ml^−1^ using vivaspin columns and stored at −20 °C.

### Assays of biofilm formation

For Congo red binding assays, strains were cultured overnight in LB lacking salt. The next day, 5 µl of the culture was spotted onto LB agar lacking salt and supplemented with 40 µg ml^−1^ of Congo red. The agar plates were then incubated at 37 °C overnight. The morphology and colour of colonies were recorded by digital photography. The experiments were done at least three times to check that colony phenotypes were reproducible, and images shown are representative. The crystal violet assay described by Baugh *et al*. [35] was used to quantify biofilm production. Two independent overnight cultures per strain were diluted in LB to an OD_600_ of 0.1. A 200 µl aliquot was added to a flat-bottomed 96-well microtitre plate, with four replicate wells per culture. The plate was incubated at 30 °C for 48 h. Wells were washed with water to remove unattached cells and 200 µl of 0.1 % (*w/v*) crystal violet was added for 15 min. Wells were then washed with water again to remove unbound crystal violet and 200 µl of 70 % ethanol was added to solubilise the retained crystal violet. The A_600_ was then measured using a CLARIOstar plate reader (BMG Labtech) to give a quantitative measure of biofilm formation.

## Results

### Genome-wide distribution of MarA, SoxS, Rob and RamA in *Salmonella* Typhimurium

**Fig. 1.**
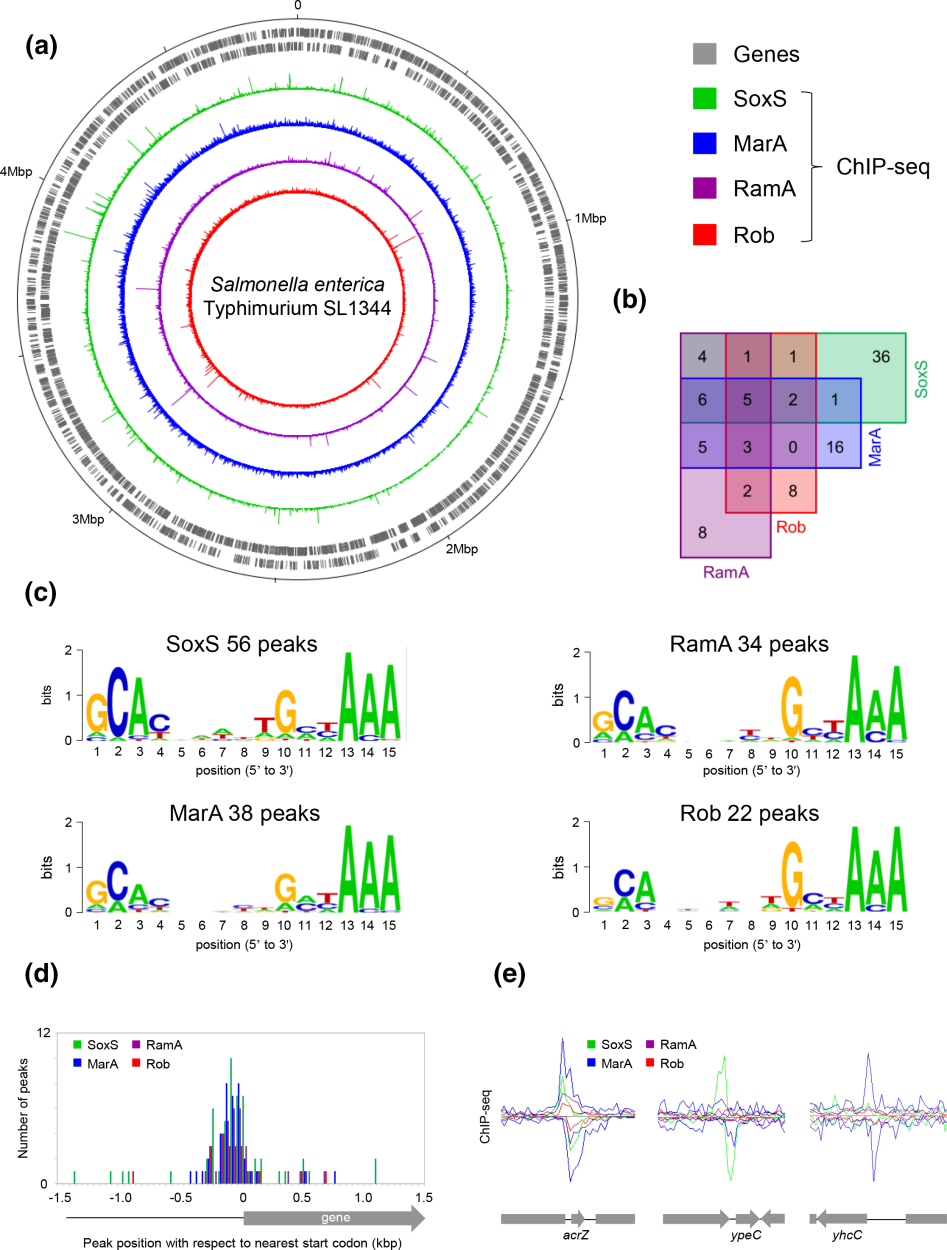
Genome-wide distribution of MarA, SoxS, Rob and RamA in *Salmonella* Typhimurium. (a) Distribution of SoxS, MarA, RamA and Rob across the *S*. Typhimurium chromosome. Genes are shown as grey lines (outer two tracks) and ChIP-seq binding profiles for SoxS, MarA, RamA and Rob are in green, blue, purple and red respectively. (b) Venn diagram indicating the number of overlapping binding peaks for SoxS, MarA, RamA and Rob. Colour coding as in panel (a). (c) DNA sequence logos generated by aligning binding sites for the indicated transcription factor recovered from the indicated number of ChIP-seq binding peaks. (d) Position of binding peak centres with respect to the nearest gene start codon. Data are shown individually for the different regulators and coloured as in panel (a). (e) Example binding peaks at three different genomic loci. Genes are shown as grey block arrows and the ChIP-seq binding signal is presented as a line graph indicating the depth of reads mapping to the top or bottom strand.

The *S*. Typhimurium SL1344 genome consists of a 4 878 012 bp chromosome and three plasmids (pCol1B9, pRSF1010 and pSLT) [36]. We used ChIP-seq to map the distribution of MarA, SoxS, Rob and RamA across each of these DNA molecules. To facilitate this, genes encoding each of the regulatory factors were cloned in derivatives of plasmid pAM. Each version of the plasmid encodes a 3×FLAG or 8×Myc tag. These sequences can be fused to the 3′ or 5′ end of the cloned gene. In preliminary experiments, we tested the various fusion proteins for utility in ChIP-seq assays. We determined that N-terminal 3×FLAG fusions were most suitable for ChIP-seq experiments with SoxS, Rob and RamA. An N-terminal fusion with MarA was also favoured with the 8×Myc tag best in preliminary tests. Alternative tagging strategies either failed completely or produced poor ChIP-seq profiles. The chromosomal MarA (blue), SoxS (green), Rob (red) and RamA (purple) binding profiles are shown in Fig. 1(a). In the schematic, genes on each strand are shown as grey lines. A detailed list of binding targets is provided in Table 1. A total of 38, 56, 22 and 34 binding peaks were identified for MarA, SoxS, Rob and RamA, respectively. We speculate that the comparatively small number of binding peaks for Rob may indicate sequestration in aggregates in the absence of dipyridyl [16]. The overlap between peak positions for each protein is shown by the Venn diagram in Fig. 1(b). Of the four factors, SoxS has the most distinct regulon, with only 20 of the 52 peaks overlapping the binding signal for at least one of the other three regulators. Conversely, RamA binding was the least distinct: 26 of the 34 RamA peaks overlap with those of MarA, SoxS or Rob. We used MEME to identify the consensus DNA binding motif for the four sets of peaks; as expected, these were nearly indistinguishable (Fig. 1c). Consistent with our prior analysis of MarA binding across the *E. coli* genome [4], we found that binding sites for all four factors occurred most frequently within non-coding DNA just upstream of gene start codons (Fig. 1d). Example binding profiles are shown in Fig. 1(e). The *acrZ* gene regulatory region was a target for all four factors, whilst *ypeC* and *yhcC* each had binding peaks for only one factor.

**Table 1.** Binding targets for MarA, SoxS, Rob and RamA identified using ChIP-seq

Binding protein	Peak	Site	Gene(s)*	*P*-value	Site (5′ to 3′)	* E. coli* MarA^a^ or SoxS^b^†
		*S.* Typhimurium SL1344 chromosome				
MarA, RamA	52 564	52 620	*rpsT<>yaaY*	2.04E-04	AAATCCATTGACAAA	
MarA, Rob, SoxS	134 070	134 036	*leuL<>leuO*	1.38E-06	GCACAATTAGCTAAA	yes^a^
MarA, RamA, Rob, SoxS	156 902	156 874	*lpxC*	1.21E-04	GCTCTTTGTGCTAAA	yes^b^
RamA	174 940	174 928	*aroP<>pdhR*	1.21E-04	GCATTCGCGGCCACA	
MarA	202 096	202 029	*yadF<>yadG*	2.40E-04	GCACTATGGTCAAAA	
SoxS	424 246	nd	*hemB<>yaiU*	nd	nd	
SoxS	435 010	435 025	0377	1.00E-04	GAACCACCAGGAAAA	
MarA	482 680	482 714	*phnS*	5.57E-04	GCTTATATGACAAAA	
MarA, Rob, SoxS	497 974	498 029	*cyoA*	9.09E-06	CCATCAATTGATAAA	
SoxS	508 034	507985	*cypD*	4.48E-04	GCCTATTGTGACAAG	
SoxS	515 659	515 659	0452*<>ybaO*	3.94E-07	GCACAAAATGATAAA	yes^a,b^
MarA, RamA, SoxS	524 017	524 010	*ybaZ*	5.61E-05	GCCCTGCCAGCTACA	
MarA, RamA, SoxS	533 256	533 255	*acrA < > aefA*	5.61E-05	GCACGAAAAACCAAA	yes^b^
MarA, RamA	539 646	539 652	*priC < > apt*	4.16E-04	GCGCAGGCGGTCAAA	
SoxS	568 176	568 173	(*ybbP*)	2.23E-06	GCACAATCGGATAAA	
Rob	598 569	598 459	*ppiB<>cysS*	4.57E-05	GAACAGGATGCAAAA	
MarA	692 417	nd	(*cspE*)	nd	nd	
MarA	711 235	711 324	*leuS<>0637*	3.57E-04	GCCCATAAAAATAAA	
SoxS	757 147	757 191	*fldA*	2.38E-05	GCACGCTCTGCTACA	yes^b^
RamA, SoxS	781 807	781 785	0698	2.04E-04	ACAAAAATGGATACA	
SoxS	792 015	792 022	0709	2.59E-06	GCATCGCGTGCTAAA	
MarA, RamA, Rob, SoxS	844 579	844 497	*modE<>acrZ*	6.42E-04	CCAGCTCCTGGTAAA	yes^a,b^
RamA	898 723	898 691	*ybiF<>ompX*	3.06E-04	AAACGTTCTGTTACA	
Rob	1 014 845	1 014 820	*pflB*	5.61E-05	GCAGCAATGGCCAAA	
MarA	1 068 935	1 069 003	0962	2.04E-04	GAATATACCACCAAA	
SoxS	1 186 956	1 186 937	*csgD<>csgB*	1.87E-04	GCACAAAGACAAAAA	
RamA SoxS	1 292 953	1 292 918	STnc1210	7.99E-06	GCACAGATCGCTAAA	
MarA, RamA, Rob,	1 416 444	1 416 555	*lppB*	2.61E-04	GCATTCCCATCAAAA	
MarA, RamA	1 465 849	1 465 776	*purR<>ynhF*	6.88E-04	GCCCGTTTCGCTACA	
MarA	1 466 921	1 466 965	*sodB*	3.86E-04	AAACGACAGGATAAA	
RamA	1 550 776	nd	(*STnc560*)	nd	nd	yes^a^
RamA, Rob, SoxS	1 554 865	1 554 877	*marR<>marC*	4.65E-06	CCACGATTTGCTAAA	
SoxS	1 603 120	1 603 084	(*sfcA*)	8.27E-07	GCACATTCTGCAAAA	
SoxS	1 650 151	1 650 167	*yncJ*	7.00E-06	GCACTTATTGACAAA	
SoxS	1 698 924	nd	*nifJ*	nd	nd	
MarA, RamA, Rob	2 064 839	nd	(1958)	nd	nd	
SoxS	2 097 359	2 097 370	(*cobU*)	1.32E-05	GCACGTAGTGGTAAA	
SoxS	2 145 247	2 145 230	(*yeeY*)	2.59E-06	GCATTATTTGCTAAA	
MarA, RamA SoxS	2 364 593	2 364 591	*ompC<>micF*	8.48E-08	GCACTGAATGATAAA	yes^a,b^
SoxS	2 520 920	2 520 938	*2373<>ypeC*	3.23E-07	GCATTTTTTGCTAAA	yes^a,b^
MarA	2 594 769	2 594 825	*ypfM<>yffB*	3.70E-05	ACCCAATTTGATAAA	
MarA, RamA	2 600 650	2 600 628	*purC*	7.88E-04	GAAATAGCGGTTAAA	
MarA, RamA, SoxS	2 623 708	2 623 719	*guaB<>xseA*	1.12E-08	GCACTATTTGCAAAA	yes^a^
MarA, RamA, SoxS	2 759 658	n.d.	(*isrJ*)	nd	nd	
RamA, SoxS	2 763 581	2 763 538	2584	2.23E-06	GCACTTTTTGCAAAA	
RamA	2 767 331	2 767 262	(*gpP*)	4.16E-04	GCAGAAGTTGCTAAC	
Rob	2 768 467	2 768 394	cIIa<>2594	3.86E-04	GACTTGTTGGTAAAA	
RamA, Rob	2 855 168	nd	2664><2665	nd	nd	
SoxS	2 891 166	2 891 166	(2712)	1.62E-06	GCACATAGTGATAAA	
SoxS	2 984 058	2 984 075	(*emrR*)	1.17E-06	GCACTTCTTGCAAAA	
SoxS	2 999 313	2 999 307	*ygaD*	1.90E-06	GCACAAACTGAAACA	
RamA	3 121 825	3 121 821	(*pyrG*)	2.82E-04	ACCCCGCCGGTCACA	
MarA, RamA, Rob, SoxS	3 156 896	3 156 839	(*gcvB*)	2.21E-04	CAACCGTAAGCCAAA	
MarA, SoxS	3 219 202	3 219 230	3014*<>idi*	7.36E-04	AAAGGCATTACCAAA	
Rob	3 242 952	nd	*ygfA*	nd	nd	
MarA	3 277 271	3 277 264	(*yggJ*)	3.06E-04	GAACGTCTGAACAAA	
SoxS	3 369 166	3 369 096	*nudF<>tolC*	1.10E-04	GCAATAATGATTAAA	yes^a^
MarA	3 511 816	3 511 923	*yhbL<>acrZ*	1.69E-05	GCAAACGCGGAAAAA	yes^a,b^
MarA	3 515 330	3 515 447	*yhcC<>gltB*	1.17E-05	GCAAACGCTGAAAAA	
SoxS	3 550 108	3 550 003	*yhcN*	3.23E-07	GCATGATTTGCCAAA	
SoxS	3 570 791	3 570 803	(3351)	1.50E-05	GCATAGCTGGTTAAA	
SoxS	3 581 551	3 581 481	*acrE*	2.59E-06	GCAATTAATGCCAAA	
SoxS	3 602 846	3 602 832	*sapG><*3378	4.03E-06	ACACCCACTGCCAAA	
MarA, RamA, Rob, SoxS	3 618 132	nd	*rpsJ<>hopD*	nd	nd	
MarA	3 758 162	3 758 200	*rpoH*	1.03E-05	TCACTGTCTGATAAA	
SoxS	3 795 560	3 795 552	(3566)	7.99E-06	GCATTTTTAGAAAAA	
SoxS	3 801 691	3 801 721	*yhjB<>yhjC*	1.71E-07	GCACATTTTGTTAAA	
MarA	3 829 638	nd	STnc710	nd	nd	
MarA, RamA	3 838 439	nd	(3597)	nd	nd	
MarA	3 838 473	nd	*dppA*	nd	nd	
MarA, RamA, Rob	3 857 661	nd	*cspA*	nd	nd	
SoxS	3 867 491	3 867 462	*yiaB*	5.34E-06	GCATCGCCGGACAAA	
SoxS	3 878 460	3 878 455	*yiaM*	4.77E-07	GCACAAAATGAAAAA	
SoxS	3 879 412	3 879 302	(3635)	5.19E-04	GCATTGATTTCCAAC	
Rob	3 926 576	3 926 691	*kbl<>rfaD*	1.50E-05	GCCCTGAATGATAAA	
RamA	3 962 578	3 962 520	*gltS<>yicH*	2.21E-04	GACCAGATGGTAAAA	
SoxS	3 974 136	3 974 110	*rmbA*	2.04E-04	ACCCCACAAGCAAAA	
SoxS	3 974 754	3 974 833	*rmbA*	2.04E-04	GCATTAAGTTACAAA	
SoxS	3 975 126	3 975 123	*rmbA*	1.57E-08	GCACTATTTGCTAAA	
RamA SoxS	4 031 825	4 031 845	*hslT<>yidQ*	4.03E-06	GCACTGATTGTTAAA	
RamA	4 080 491	4 080 583	*yieG<>yieH*	1.44E-04	GCCGTCACAGTCAAA	
MarA, RamA, Rob, SoxS	4 130 272	4 130 274	*comM<>ilvX*	6.20E-05	GCAAGAATAGACAAA	
Rob	4 147 006	4 146 922	*rho*	2.67E-05	GAAGTGACGGATAAA	
SoxS	4 167 473	4 167 472	(*hemC*)	4.65E-06	GCACATTATGTCAAA	
MarA	4 196 382	4 196 381	*dlhH<>udp*	2.40E-04	GCTTCTTCTGACAAA	
MarA	4 198 397	4 198 306	(*yigN*)	1.00E-04	GCCCGAACTGATAAC	
RamA, Rob	4 230 601	4 230 569	*polA><engB*	2.04E-04	AAATATTCAGCCAAA	
Rob	4 231 620	4 231 532	*engB<>csrC*	3.57E-04	TAATTGTCTGAAAAA	
SoxS	4 248 761	4 248 789	(*yihP*)	2.23E-06	GCACGCAAGGATAAA	
SoxS	4 287 932	4 287 842	4003*<>sodA*	7.00E-06	GCATCCGCTGAAAAA	yes^b^
SoxS	4 314 627	4 314 649	*fpr*	7.54E-05	GCTCTAACTAACAAA	yes^b^
MarA	4 497 504	4 497 448	*ssb*	2.38E-05	GCATCTTCAGCTAAA	
SoxS	4 666 487	4 666 601	*msrA<>ytfM*	3.86E-04	CCACCCCTGGAAAAA	
SoxS	4 673 449	4 673 443	(4345)	1.17E-05	GCACCAGCCGACAAA	
Rob	4 720 090	4 720 013	*treR<>mgtA*	1.00E-04	GCCATAATTGCCACA	
Rob, SoxS	4 844 268	4 844 383	*deoB*	1.57E-04	ACACTCTGGGCCACA	yes^a^
MarA, RamA, SoxS	4 851 868	4 851 909	4502	6.92E-07	GCACAAATAGTTAAA	
RamA	4 864 34	4 864 193	*rob<>creA*	1.62E-06	ACACTGAATGCTAAA	yes^b^
			S. *Typhimurium plasmid pSLT*			
SoxS	74 381	74 501	P1_0081	3.10E-09	GCACAAATTGCTAAA	
SoxS	78 949	79 068	*pefB*	2.80E-05	GCACAAAAAATCAAA	

*Binding sites were located between divergent genes (<>) convergent genes (<>) or upstream of genes. Genes in parenthesis indicate intragenic binding sites. Where numbers are provided, the gene remains unnamed and the number is an abbreviation of the locus tag (e.g. SL1344_0377 is shown as 0377).

†Identified as targets for MarA or SoxS, by ChIP-seq^a^ or ChIP-exo^b^ respectively, in *E. coli*.

### Conservation of the regulatory network in other bacteria

**Fig. 2.**
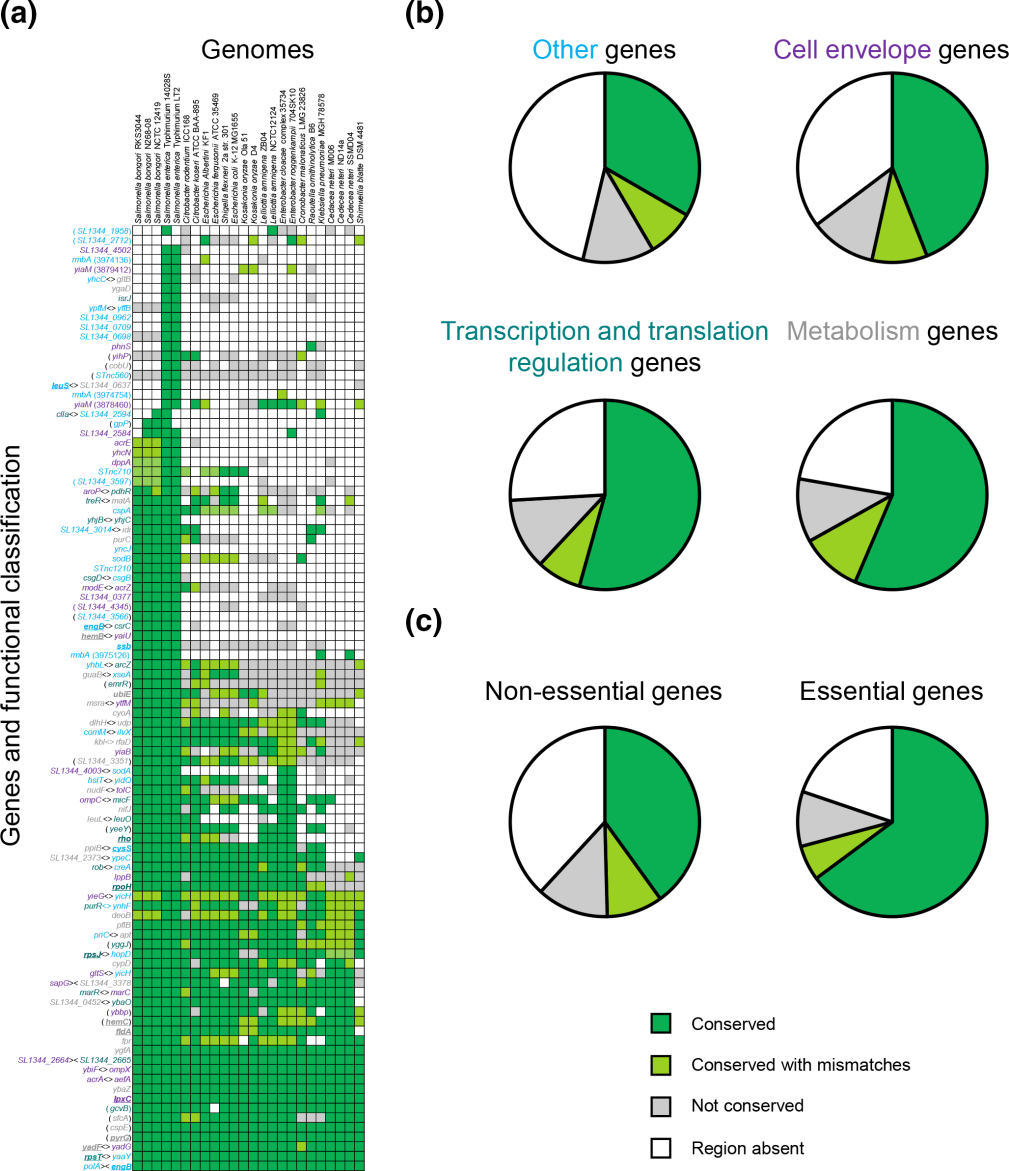
Function and conservation of MarA, SoxS, Rob and RamA targets. (a) The heatmap lists binding targets for MarA, SoxS, Rob or RamA in *S*. Typhimurium (*x*-axis) and species in which a MarA homologue can be identified (*y*-axis). Intersections are coloured depending on conservation of the binding site identified using ChIP-seq in this study. Essential genes [37] are underlined. Gene names are coloured according to roles in cell envelope biology (purple), gene regulation (teal) or metabolism (grey). Genes with other functions are labelled with cyan text. Note that cell colour indicates conservation of the binding target for MarA, SoxS, Rob or RamA, not conservation of the adjacent gene. (b) Each pie-chart summarizes information in the panel (a) heatmap, for different categories of genes. Each pie-chart section, coloured according to conservation of the MarA, SoxS, Rob or RamA binding sites, depicts the number of equivalent panel (a) heatmap cells for each group of genes. (c) As for panel (b), but MarA, SoxS, Rob and RamA targets are grouped according to the essentiality of adjacent genes.

We next turned our attention to understanding the conservation of binding targets and the function of adjacent genes. Fig. 2(a) lists representative bacterial species across the full diversity of organisms encoding a MarA-like protein (*x*-axis) [4]. The *y*-axis lists different binding targets with gene names coloured according to their function. Overall, genes adjacent to binding targets for MarA, SoxS, Rob and RamA, are most likely to encode factors involved in cell envelope biology (purple), gene regulation (teal) or metabolism (grey). Genes with other functions are labelled with cyan text. Where gene and species names intersect, the heatmap cell is coloured according to conservation of the identified binding site. Around one third of the binding targets are conserved in almost all genomes examined (dark or pale green squares). The most common reason for a binding target not being detected in another species is that a sequence aligning with the equivalent *S*. Typhimurium regulatory DNA is not present (white squares). For instance, the *dppA* gene is found in many enteric bacteria but the upstream regulatory DNA often has a drastically different sequence. Comparatively, it was rare for an equivalent DNA region to be found but the binding site absent (grey squares). Fig. 2(b) shows a series of pie charts summarizing Fig. 2(a) heatmap for different subsets of targets, grouped according to the function of adjacent genes. Hence, for all genes with ‘other’ functions, the pie chart depicts the percentage of the Fig. 2(a) heatmap cells in each category. Binding targets adjacent to genes encoding metabolic or gene regulatory functions are more likely to be conserved than targets at genes for cell envelope associated factors. Targets in the ‘other’ category, which includes many *S*. Typhimurium specific genes, are least likely to be conserved. We also compared binding site conservation data for essential and non-essential genes [37] (Fig. 2c). Overall, 15 of the 98 binding targets were adjacent genes judged to be essential. Such sites were almost twice as likely to be conserved than those adjacent to non-essential genes.

### Changes in global transcription start site use induced by MarA, SoxS or RamA expression

**Fig. 3.**
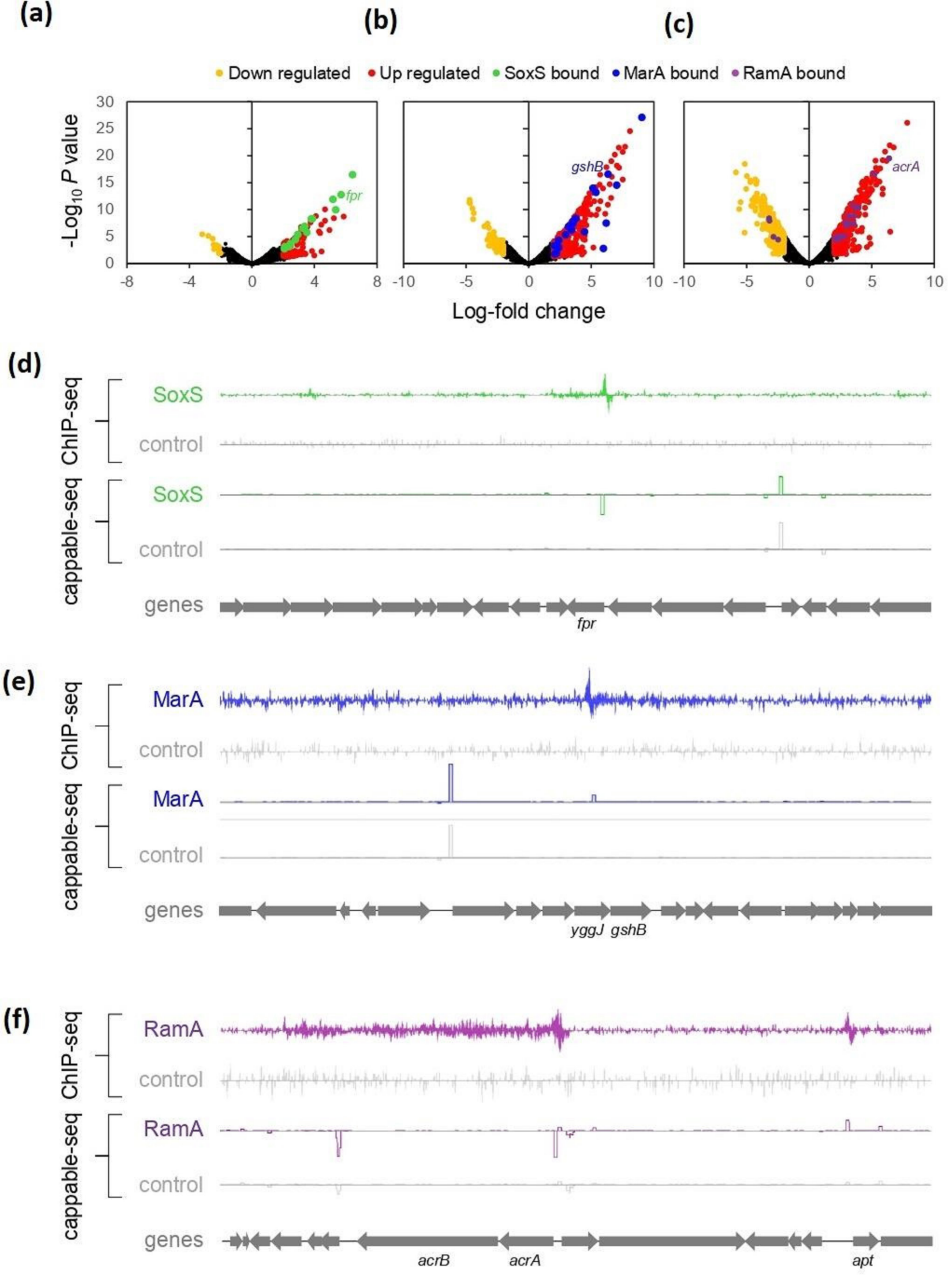
Changes in global transcription start site use resulting from expression of MarA, SoxS and RamA. (a) Volcano plot showing changes in transcription start site (TSS) use induced by expression of SoxS. Each data point represents an individual TSS and is coloured orange or red to indicate significant down or up regulation, respectively. Significant changes that coincide with binding of SoxS are in green. Black data points correspond to TSSs with no significant change. Data points corresponding to the expansions in panels (b)–(d) are labelled. (b) Volcano plot showing changes in TSS use induced by expression of MarA. Data points coloured as in panel (a) except that coincidence with MarA binding is shown in blue. (c) Volcano plot showing changes in TSS use induced by expression of RamA. Data points coloured as in panel (a) except that coincidence with RamA binding is shown in purple. (d) Activation of the *fpr* gene by SoxS. Each graph is a plot of sequencing read depth mapping to the top or bottom DNA strand. For ChIP-seq experiments, the peak indicates binding of SoxS. For cappable-seq, the 5′ boundary of the peak is a TSS. Data were generated either from cells having ectopic SoxS expression from pAMNF (green) or carrying empty pAMNF (grey). Genes are shown as block arrows. (e) Activation of the *gshB* gene by MarA. Coloured as in panel (d) except that expression of ectopic MarA corresponds to the blue traces. Note that the *gshB* promoter is located within the *yggJ* coding sequence. (f) Activation of the *acrAB* operon by RamA. Coloured as in panel (d) except that expression of ectopic RamA corresponds to the purple traces. Note that the nearby *apt* gene is also subject to direct activation by RamA.

Whilst ChIP-seq captures DNA binding events, consequences for transcription from nearby promoters are not determined. Hence, we used cappable-seq to better understand regulatory outcomes associated with expression of the MarA, SoxS or RamA fusions (Rob was excluded given the small number of unique binding peaks). Note that, whilst standard RNA-seq maps overall transcript abundance, cappable-seq targets the RNA 5′ end. Consequently, as well as providing a measure of transcriptional activity, cappable-seq identifies transcription start sites (TSSs) [29]. Fig. 3(a–c) show changes in the cappable-seq signal, for each TSS, induced by expression of the SoxS, MarA or RamA fusion proteins. Significant increases (red) and decreases (orange) in signal are indicated. Changes that coincide with a SoxS, MarA or RamA binding peaks are coloured green, blue or purple, respectively. There are two notable features of the volcano plots. First, most of the significant changes in transcription do not coincide with direct SoxS, MarA or RamA binding, indicating that regulation is likely to be indirect. Second, expression of the RamA fusion protein induced the most changes in TSS use. Fig. 3(d–f) show transcriptional changes associated with direct binding of SoxS, MarA or RamA, which activate *fpr*, *gshB* and *acrAB*, respectively.

### SoxS, MarA and RamA target housekeeping RNA polymerase promoters

We next focused our attention on understanding the properties of promoters targeted by SoxS, MarA or RamA. As a starting point, we made an inventory of regulatory regions where the binding site identified by ChIP-seq could be unambiguously assigned to a TSS identified by cappable-seq. Hence, we excluded regulatory regions with multiple TSSs unless the TSS subject to regulation was clear (i.e. the cappable-seq signal for a specific TSS changed upon expression of the regulator). Similarly, to minimize erroneous designations, we excluded regions with either no, or multiple, potential binding sites for SoxS, MarA or RamA. Taking this cautious approach, we allocated TSSs to 36 of the binding sites identified by ChIP-seq. The regulatory region sequences are shown in Fig. S1, with TSSs (green) and binding motifs for SoxS, MarA or RamA (blue) highlighted. We searched DNA sequences upstream of the TSSs for motifs indicative of RNA polymerase σ factor specificity. In all cases, we identified appropriately positioned DNA elements upstream of transcription start sites. Universally, these matched the sequences recognized by the house keeping σ^70^ factor (highlighted red in Fig. S1). Whilst promoters dependent on σ^70^ can often also be used by σ^38^, during periods of starvation, we did not identify any motifs associated with binding of alternative σ factors.

### Architecture of promoters targeted by SoxS, MarA and RamA

**Fig. 4.**
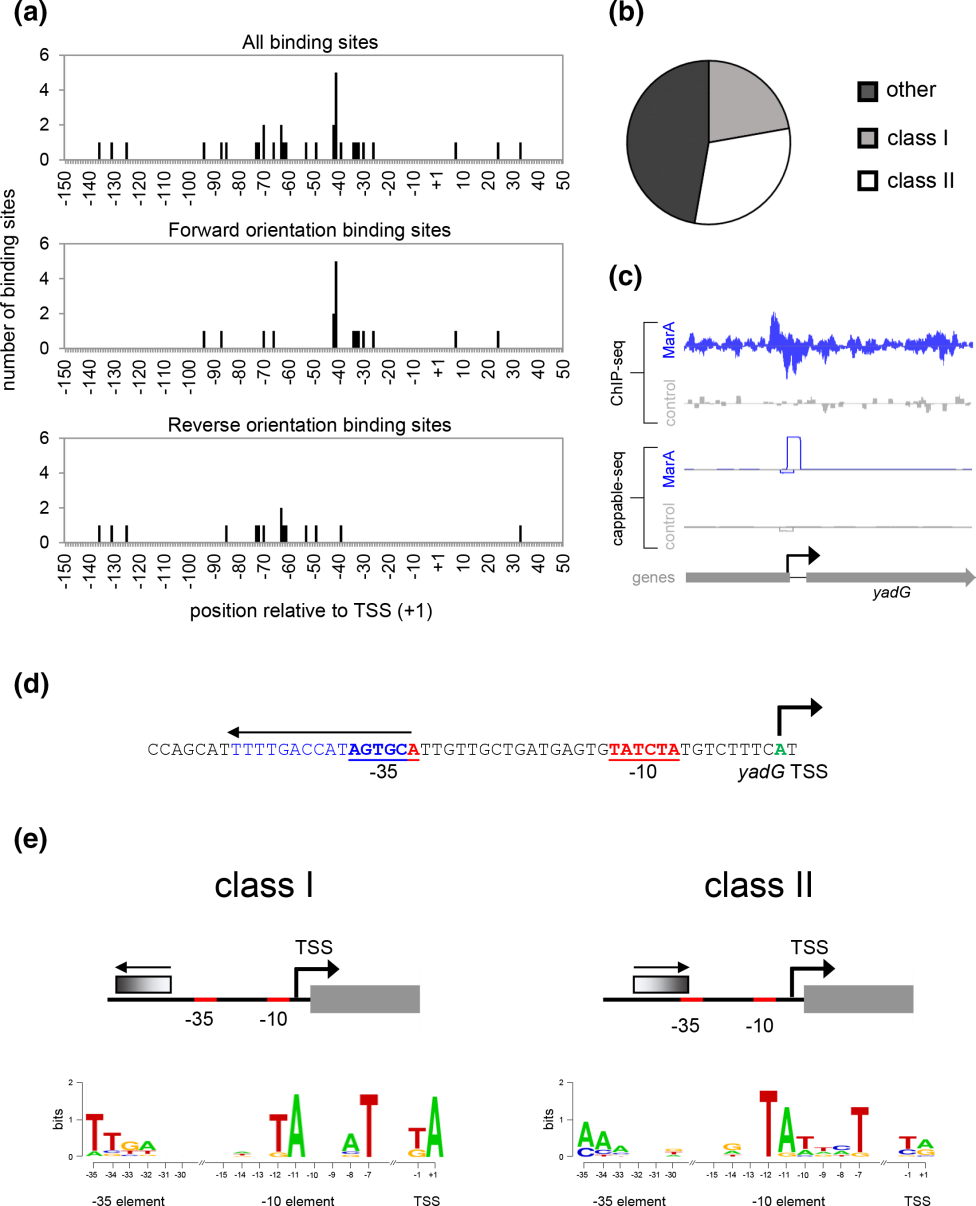
Architecture of promoters targeted by MarA, SoxS and RamA. (a) Position of binding sites for MarA, SoxS and RamA with respect to transcription start sites (TSSs). The histograms show data for all binding sites (top) or only those in the forward (middle) and reverse (bottom) orientations. (b) The pie-chart illustrates the percentages of promoters with class I, class II or another organization. (c) Activation of *yadG* by MarA. Each graph is a plot of sequencing read depth mapping to the top or bottom DNA strand. For ChIP-seq experiments, the peak indicates binding of MarA. For cappable-seq, the 5′ boundary of the peak is a TSS. Data were generated either from cells having ectopic MarA expression from pAMNM (blue) or carrying empty pAMNF (grey). Genes are shown as block arrows. (d) Architecture of the *yadG* promoter region. Core promoter elements for recognition by the RNA polymerase σ^70^ subunit are underlined. The MarA binding site is in blue. The TSS is in green and identified by a bent arrow. (e) Sequence properties of class I and class II promoters. The schematic diagrams illustrate organization of each promoter class. The DNA logos represent the sequences of the promoters classified in panel (b).

Previously, *E. coli* promoters activated by MarA have previously been divided into two classes [38]. At class I promoters, the MarA binding site is in the reverse orientation and positioned distal to the core promoter elements. Conversely, at class II promoters, the binding site is in the forward orientation and overlaps the promoter −35 element for σ^70^ binding. Targets for SoxS and RamA conform to the same organizational rules [39, 40]. Hence, we determined the position and orientation of each binding site for MarA, SoxS or RamA with respect to TSSs and core promoter elements. Fig. 4(a) (top panel) illustrates the position of all binding sites with respect to the assigned TSS. The three most common binding positions are grouped around 41 bp, 62 bp and 72 bp upstream of the TSS. The same analysis was applied to only those binding sites in the forward (middle panel) or reverse (bottom panel) orientation. The cluster of sites near position −41 were primarily in the forward orientation. Conversely, sites near position −62 and −72 were usually in the reverse orientation. Overall, there was a trend for reverse orientation binding sites to be located further away from the core promoter elements (compare middle and bottom panels). Of the 36 regions assessed, 8 and 11 obeyed class I and class II position and orientation rules, respectively. The remaining 17 promoters had SoxS, MarA or RamA binding sites in other configurations (Fig. 4b). For example, the *yadG* promoter is bound, and activated, by MarA (Fig. 4c). Inspection of the DNA sequence reveals an excellent match to the consensus binding site for MarA overlapping the promoter −35 element in the reverse orientation (Fig. 4d). Hence, in this instance, the MarA binding site is in a class II position but not orientation.

### Class I and class II promoters have different sequence properties

To better understand the sequence properties of class I and class II promoters we aligned the core promoter elements for RNA polymerase binding to generate DNA sequence logos (Fig. 4e). Whilst promoters in both classes had canonical TSSs and promoter −10 elements, the sequence of the −35 hexamer was completely different. Class I promoters matched the consensus −35 element sequence, 5′-TTGACA-3′, at an average of 4.3 positions. Conversely, consistent with an overlapping site for MarA, SoxS, or RamA, none of the class II promoters matched the −35 hexamer at more than three positions. Half did not match the −35 hexamer at any position. The average number of matches was 1.9. The occurrence of extended −10 elements, characterized by a 5′-TG-3′ motif at promoter positions −14 and −15, was similar for both promoter classes (Fig. S1).

### The *csgDEFG* operon is a direct target for SoxS

**Fig. 5.**
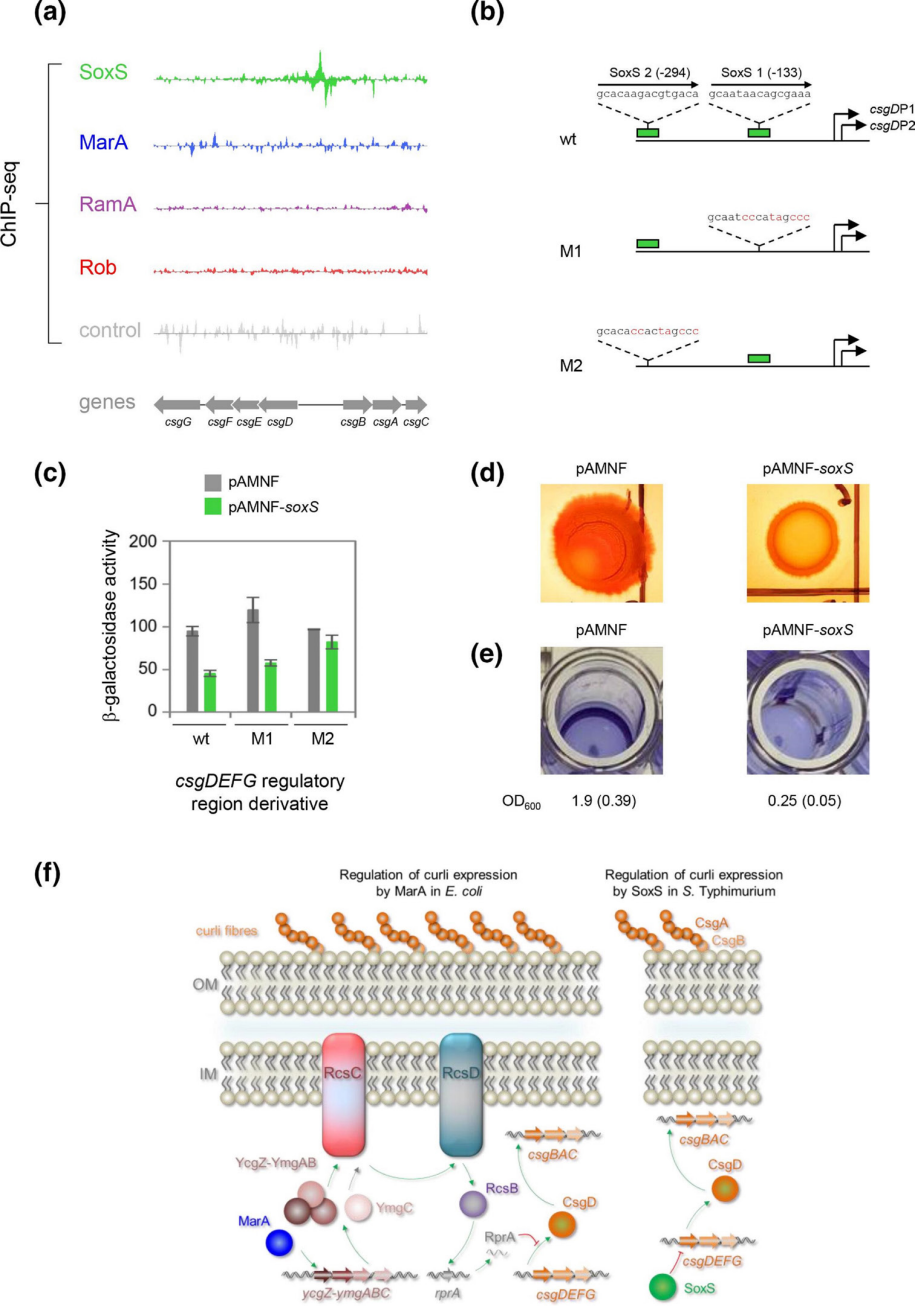
Direct repression of *csgDEFG* expression and biofilm formation by SoxS. (a) SoxS binds to the *csgDEFG* regulatory region *in vivo*. ChIP-seq binding signals for SoxS, MarA, RamA and Rob at the *csgD* locus. Graphs show sequencing read depth for the top and bottom DNA strand. Genes are shown as block arrows. (b) Organization of the *csgDEFG* regulatory region. The schematics indicate the positions of likely TSSs deduced from comparison with the known *csgD* P1 and P2 promoters in *E. coli *. Numbering is with respect to the *csgD* P1 TSS. Two potential binding sites for SoxS, identified by ChIP-seq, are labelled. Mutations designed to remove each binding site in the M1 and M2 DNA fragments are in red text. (c) The bar charts show the mean β-galactosidase activity from three independent replicates. Error bars show standard deviation. (d) Macrocolonies formed by the indicated strains on agar plates containing Congo red dye. Larger images are in Fig. 3. (e) Crystal violet staining of biofilms formed by the indicated strains. Each image shows a well from a microtitre plate. Prior to images being captured, cells were stained with crystal violet dye, planktonic cells were removed and the remaining dye was solubilized. The OD_600_ values, from three independent experiments, are shown below each corresponding image. Standard deviations are shown in parenthesis. (f) Models for regulation of curli fibre expression in *Escherichia coli* and *Salmonella* Typhimurium. Genes are shown as block arrows and encoded proteins by spheres. Positive and negative regulatory interactions are shown by green arrows and red barred lines, respectively. The inner and outer membranes (IM and OM, respectively) are labelled and periplasm is shaded pale blue. The cell wall is not shown for simplicity. The model for *E. coli* (left) is based on our previous study whilst the regulatory pathway for *S*. Typhimurium is based on the current work. Whilst *S*. Typhimurium lacks *ycgZ-ymgABC*, we do not exclude the possibility that *csgDEFG* may also be subject to direct control by SoxS in *E. coli *.

We next sought to better understand regulatory regions, bound by SoxS, MarA or RamA, that do not conform to class I or class II rules. Our attention turned to the intergenic region upstream of the *csgDEFG* operon, that exhibits a prominent peak for binding of SoxS in our ChIP-seq experiments (Fig. 5a). A schematic of the wild-type region is shown in Fig. 5(b) (top panel), and the full sequence is shown in Fig. S2. Two potential binding sites for SoxS, labelled site I and site II, are highlighted.

### The csgD promoter is directly repressed by SoxS *in vivo*

The *csgDEFG* mRNA 5' was not detected in our cappable-seq analysis. This may be because the RNA is present at low levels. Hence, we could not deduce any regulatory effect of SoxS. As an alternative approach, we cloned the *csgDEFG* regulatory DNA upstream of *lacZ* in plasmid pRW50T. We also made derivatives with mutations in each of the two potential SoxS sites (named M1 and M2, respectively, Fig. 5b). The DNA constructs were transferred into *S*. Typhimurium SL1344, with or without pAM expressing *soxS*, by conjugation. We then measured β-galactosidase activity of cell lysates. The data are shown in Fig. 5(c) (solid bars). Expression of SoxS significantly reduced *lacZ* expression from the wild-type DNA fragment. The M1 fragment remained subject to such a regulation, whilst the M2 fragment was freed from control by SoxS.

### Expression of SoxS reduces curli fibre production and biofilm formation

CsgD is required for the production of curli fibres and for biofilm formation. Production of curli can be monitored using Congo red dye that binds the fibres. Fig. 5(d) shows *S*. Typhimurium macrocolonies grown on agar plates containing Congo red (see Fig. S3 for larger images). Wild-type cells carrying empty pAMNF, form red colonies with a nonuniform rough/wrinkled surface Ectopic expression of SoxS from plasmid pAMNF resulted in pale colonies with a uniformly smooth surface, consistent with repression of *csgD* by SoxS. We also monitored biofilm formation directly by staining with crystal violet. Briefly, in these assays, liquid cultures are incubated overnight in a polystyrene microtitre plate. The next day, after removing planktonic microbes, cells attached to the solid surface can be detected by crystal violet staining. The results are shown in Fig. 5(e). The images depict representative wells and quantification of the signal from three independent replicates is shown below each panel. Expression of SoxS reduced biofilm production eightfold.

## Conclusions

Of the *S*. Typhimurium MarA, SoxS, Rob and RamA targets identified here, 34 map to promoters where the binding site sequence is conserved in at least 75 % of organisms encoding a MarA-like protein (Fig. 2a). This represents a conserved core regulon predominantly encoding genes involved in the control of gene expression, metabolism and cell envelope biology. Most likely, this signifies a universal strategy, used by many organisms, to survive harmful conditions. Conversely, many binding targets specific to *Salmonella* sp. are adjacent to genes encoding more diverse functions. Such ancillary regulon components likely optimize stress responses in a species-specific manner. Interestingly, ten genes targeted by MarA, SoxS, Rob and RamA encode other transcription factors. Hence, depending on the conservation of downstream targets, the indirect regulatory effects of MarA, SoxS, Rob and RamA could differ markedly between organisms. We note that our strategy of constitutively expressing the different regulatory factors, to induced changes in transcription, likely avoids pleiotropic responses to the stress conditions that usually induce MarA, SoxS, Rob and RamA expression [41].

In our conditions, all promoters targeted by MarA, SoxS, Rob or RamA had sequence properties consistent with σ^70^ dependence. Indeed, to our knowledge, no promoters dependent on alternative σ factors are controlled by these regulators. This appears true even when a promoter is recognized by more than one σ factor; the *E. coli ycgZ-ymgABC* promoter is recognized by σ^70^ and σ^38^, but MarA only activates σ^70^ dependent transcription [34]. Together, these observations are consistent with MarA, SoxS, Rob and RamA mediating an ‘emergency’ response resulting from sudden stress in an otherwise favourable environment. Even so, alternative σ factors are likely involved in the downstream indirect control of genes by MarA, SoxS, Rob and RamA. Notably, *rpoH*, encoding the alternative σ^32^ factor, resides in the core regulon (Fig. 2a). Many regulatory targets identified here influence the cell envelope (Fig. 2), and the transcriptional response to σ^32^ also alters and protects the cell membrane [42]. Indeed, σ^32^ itself is membrane-associated [43].

Overall, half of the MarA, SoxS and RamA targets to which we could assign a TSS could be designated as class I or class II promoters (Fig. 4b). With respect to their overall sequence properties, class II promoters lack a recognizable promoter −35 element (Fig. 4e). This is likely a consequence of the need to accommodate an overlapping binding site for MarA, SoxS or RamA. Furthermore, this suggests that binding of the activator takes precedent over −35 element recognition by the σ factor. Consistent with this, recent structural analysis of the class II *micF* promoter, in complex with Rob (or SoxS) and RNA polymerase, revealed displacement of σ^70^ from the promoter −35 element [15, 39]. By contrast, there is good conservation of the −35 sequence at class I promoters. Presumably, this results in tighter basal binding of RNA polymerase. Taken together, these observations may imply that binding of MarA, SoxS or RamA in a class II position results in more extensive contacts with RNA polymerase to compensate for the lack of a −35 hexamer. That not all promoters exhibit class I or class II architecture is not without precedent [24]. For instance, in *E. coli *, the marbox at the *ycgZ-ymgABC* promoter is in a class I position but the reverse orientation [34]. Similarly, the *zwf* promoter does not match the rules for class I or class II organization [24].

The *S*. Typhimurium *csgDEFG* regulatory region cannot be classified according to conventional class I or class II rules; SoxS binds 294 bp upstream of the *csgD* P1 TSS to repress transcription (Fig. 5). Given the position of the SoxS site, it is unlikely that binding of SoxS directly hinders promoter recognition by RNA polymerase. More likely, SoxS interferes with the action of an as-yet-undefined activator. Consistent with repression of *csgDEFG*, expression of SoxS represses curli fibre production and biofilm formation (Fig. 5d, e). Previously, working with *E. coli *, we identified a mechanism for MarA-mediated repression of biofilm production [34]. Briefly, MarA activates expression of *ycgZ-ymgABC* and, via a downstream regulatory cascade, represses *csgDEFG*. In *S*. Typhimurium, the *ycgZ-ymgABC* operon is absent. Hence, control of biofilm production is direct. Fig. 5(f) compares models for control of biofilm production by MarA and SoxS in *S*. Typhimurium and *E. coli *, respectively. Taken together, our findings are consistent with both organisms favouring short-term survival strategies (e.g. increased efflux and reduced membrane permeability) upon expression of MarA or SoxS, rather than *de novo* biofilm formation.

## Supplementary Data

Supplementary material 1
