# Supplementary material for: Mapping direct and indirect MarA/SoxS/Rob/RamA regulons in Salmonella Typhimurium reveals repression of csgD and biofilm formation
Source: Microbiology (Reading). 2023 May 19;169(5):001330. doi: 10.1099/mic.0.001330 (PMC10268841; doi:10.1099/mic.0.001330)
Supplement: Supplementary material 1 [file mic-169-1330-s001.pdf]

Figure S1

Class I promoters

```
acrA     CGTGTCTCCAGCGCTTGTTGTTTGGTTTTTCGTGCCATATGTCGGTGAATTTACAGGCGTTAGATTTACATACATTTATGGATGTATGTACCATAGCACGACGATAATATAAACGCAGC
fldA     AGTCTGAAAATAAGTTTTGTTTCATATTTCGTGTAGCAGAGCGTGCAGTCCCGCGTGATTGCTGTTTCCGCATATCTGCCTGTTCTGCTATGATTGCCCTTATCCGTGGGCAATTCGCC
sodB     GTGATGACGCCTAAAACAATTCGCTAATATTTTATCCTGTCGTTTAACGACAGGATAACTTCTTCTTTTGCGCCACCGCTCAATTTGCTACCCTATCCTTACGCGCACCAGGGGTATTG
marR     AAGTTATCACAGCACAATACCCCGGACGCCTTTTAGCAAATCGTGGCATCGGCCAATTCATTTAGTTGACTTATACTTGCCTGGGCAATAGTATCTGACGAAATTAATTACTTGCCGGG
ypeC     ACGCTATTCTGGAGGGCCGGTTTTAGCAAAAAATGCTCTCCTTCATGATTTAAGAGTTAAGGCGATGAAACAAATCTTTACTGCTTTGTAATATTGCATTAACAGGCCGGCGACGGCTC
2584     GTTTTACCGCCTAAAATCTGATATGAAACAACATGCTAGCTTTTGCAAAAAGTGCTATTCACCCCTTGAATATTCTTTCTAACAGGTATACTGTGTTTATATACAGTGGTTGAATGTAG
fpr      TCCGGATGTGCAGGCGCATATTCTCTAGCGCATTTGTTAGTTAGAGCCTTCCAGCCTCGCGGGGCTGGAAATCTTTCTCCTGACGAACGAAGATAAGGTTAGCGATTCAGAACAGGAGA
ybaO     TTGCTACTCATCATTAATATCCATTTATCATTTTGTGCCAAGAGTAGCGAAAAGTACGGAAAAAATTGTTGCTATTTGACCTTTAAATTACAATGTAAGGAGAAAAATATTCTCTATGG
```

Class II promoters

```
lpxC      CATTCCTGCGTAAGCAAGCTGATTAAGAATTAGCTGGAATTTGGGATTCTGGGCTCTTTGTGCTAAACTGGCCCGCCGAATGTATAGTACACTTCGGTTGGATAGGTAATTTGGCGAG
0377      TGTTTCACAAAAATGATGAACGGGGAGACAAAATAGCAAAGTGTGACGTATCGCACCTCTGGAACCACCAGGAAAAAGAGAACTTGCTAAGCTAACGCGTATTTTCAGCTACTTACCG
STnc1210  TTTAGACGGGCCTTCATTGTCTGTAGCGAAAAGTATCCCGTCATGCTTGTAGGCACAGATCGCTAAATATTATAGCCCTGACCTCTATATTATCCCGTGCCACTGACCTCGTCGCCAT
marC      TCAGATACTATTGCCCAGGCAAGTATAAGTCAACTAAATGAATTGGCCGATGCCACGATTTGCTAAAAGGCGTCCGGGGTATTGTGCTGTGATAACTTGTAACTAATTGATAATTACA
micF      AATATATTCACGAAACTTTTAAAATCAACGGGTTAAATTGATGAAATTCATAGCACTGAATGATAAAACAGAATCTTCATTCGCAACTAAAATAGTGACCGCTATCATCATTAACTTT
mltB<     CGTACGGAGAGAAAGCAAGGATGTCACTCAAAAAAACGGAAAGCACACAGCACAAACTGAAACAAACCTGTCAACTACGGCTGTGGTTAATTGTGGGTGGAGTATGTTCATTAGGAAT
rpoH      TCTTTATGTCACAAGATTTGTGCAAATTATGCACAGTGTTACATTGAACTTGTGGATAAAATCACTGTCTGATAAAAGAGTGGGTGATATTCTCGTTGCTCATCGGCTTTGGCACGGT
3635      GCCGATACGGAAAATAGTCGTAAAAGTCGCCATAGACGTTTTATAAAATAGAGTTTACTGGAGCACAAAATGAAAAATAATACCGGTTATATTATCGGTGCGTACCCTTGTGCACCCT
(yigN)    TCTGATGTTTATCCCCGTAGAACCCGCTTTCCTGCTGGCGTTAGATAAACAGCCCGAACTGATAACCGAGGCGCTGAAAAATAACATTATGCTGGTAAGCCCCACGACGCTGCTGGTG
4502      GTGTCTCTGATATTATTTAAACATAACAACATATTCTGAAAAATGAGTGAATAGCACAAATAGTTAAAACACACTCACTAACTACTATATTCTTTAAAATTAATATTATTTCCCCGCA
rob       CAGGATTAGAATAATTACCTGTCACGTTGCCTAAAAAAAGCATTTAAGCTCAAAAAAAACACTGAATGCTAAAAGAACAAAAAATGCTATTATCCTCTTACCTGATATCGGGTGCTCG
```

Other promoters

```
tolC*    CTGCTGTTTTATCACCCTTGTGGTAAATTGGTGCCAATTAAGGCTCGTATGGCACGTAACGCCAACTTTTTGCAGTGGCGGATTCTGCTAGAATCAGCAATTATTTTTACAAATTGATC
aroP     ATAAAAGGAGGTATTTACAAGTGCTTTCTTTTTACGCAACTCGTCATCACCCCTGCACAAAGCAGGTGCATTCGCGGCCACATACCATTATTCTGATCAAGACGAAAGAGTTGAAGTGA
yadG     TTTGCGCTTGCGCCAGTTTCTCAAAAAATCCGGGGTCCTCTTCCACCAGCATTTTTGACCATAGTGCATTGTTGCTGATGAGTGTATCTATGTCTTTCATGGAAGTTAACGACCTGTAA
leuL     GCACAATTAGCTAAAGTACGTATCCGGATATCGTCAACAAAATGCAATGGCGACAGAAAATAGAGTTGACATTAAACGGCATATCCAGTACCACTAAAAGCATAACGCATTCGCTGGAG
cysS     CAATGGGGCACATATAGGGGCTTGTTATAGCATAACCGTAAGCTGCGATCACCTTGCAAAGTGTGCTGCTTCGATTACGAATAATATGTATCATACGGAGATTATTACCCACACACGTC
focA     CCGTAATTGCATAAAAGCCATGCGACTTACGGGCCTATAAGCCAGGCGAGATATGATCTATATCAAATTCTCATCTATAATGCTTTGTTAGTATCTCGTCGCCGACTTAATAAAGAGAG
lpp      TTCATGCATTTCGATGCTTCTTTGAGCGAACGATCAAAAATAAGTGCATTCCCATCAAAAAAATATTCTCAACATAAAAAAGTTTGTGTAATACTTGTAACGCTACATGGAGATTAACT
yncJ     TATATTTTCTCATACAACTTATCGCACTTATTGACAAACCGTTTTCGCTAATTTGAAGTGTGGAATAATAACTCAAAGAAATTATTGGCATCATAGCCTCACGACGTAACTGGAAGGTG
yoeI     AAAATCGCCGGTTTAAAAATCAATCGCTTATGAAAAATAATTTTTTGAACGCGCTCGCATTCTCTTGTCATTTCACTTCAATTTAGGGCAGAATACGCCGCTTGCAAAAAATGACACTA
purC     AGCCAGAACGACGCGCTGGTTGCCGTCTTCCAGGCAGCGTTCAACAAGTAATTTAACAGGGCTGGAGCAATCCGGCCCTTTTTTCTGATAAGATACGCAAACGTGTGCGTCGGTACAAA
(yggJ)   GCGGTGAAAAAATGGAATTTACTATCCAGAAATCGATCGAACTGGGTGTAAGCCTCATTACGCCACTGTTCTCTGAGCGCTGCGGCGTTAAACTGGATAGTGAACGTCTGAACAAAAAG
yhcC     AATATCGTTTATTTTGCTTGATTATGCAATGGATAAAAGCCTTCCGGGACGATTGATCCAGGTCATCGCCAAAGGAGACTAAAAATGGCAGGCTTTGGCCTCTTTTTAAGAGGCGGTCT
yhcN     ATTCCATAACGTTATAAAAAATAAAATTTTTAATGTTTTTAAAAAGCAGAAACAATTAGCGTGGTATTAATTAATCGCGTGATTAGTGTATACTTAATTTTGTGATGAGGGTCACGAAA
ilvX     TATAAAAAACAAAGTACTTTTAGGCATTCACCTGCATTATCTGAAACGTGGTTAAAAAAATATCTTGTGCTATTGGCAAAACCTATGGTAACTCTTTAGGTATTCCTTCGAACAAGATG
yihO     GCTGTTTCTACAACCTCAACGAGAAGATGTACGTGCGAATTGTGGAAGAGATTGAGGCCCGGAAACATACGGTTTAACAACAATAAAATATAACGCCCTGCGGGGCGTTTTGAGAGGCG
sodA     AAACGGTTTTTTTCAGCGGATGCCGTAACGTTTATAACCCTGGAAAAAGTACGGCATTGATAATCATTTTCAATATCATTTAATTAACTATAATGAACCAACTGCTTACGCGGCGTTAA
msrA     CTATTCCCACCCCTGGAAAAAATCACACTACCGCTCCGCGGCTTAACATAAAATCAACAATTTCGATTGAAAAGCAGACGACTGCCCAAATATTTCTTATGGTTAAATCCAGATACATT
```

Figure S1: Architecture of promoters bound by SoxS, MarA or RamA corresponding to the analysis in Figure 4. The transcription start sites are in green, promoter elements in red, and binding sites identified by ChIP-seq are in blue. *We could not categorise the *tolC* regulatory region because the binding site identified by ChIP-seq (in a class II position and orientation) is in addition to a known site in a class I position and orientation.

Figure S2

acaatttgtttttcatgctgtcaccctggacctggtcgtacatagcgaaaattatc

tattaccttgttagcgacatgcgttttttgttaacgcgtcgttacgatgaagagta

tgtccgtggaaacatttttaataactcacccacgcgtggtattttgttatttaagc

tcataccaaagtgctaataaaacgatagccatgtgattttttataattgatttttg

gccacagaagatagtgtatcgcgcacctaaaaaatgaagtgttggtgtgtgttatg

ccgccatggggatgttcttatgcttcccatgtggggcaatac**gcacaagacgtgac**

acacttcgttttttttgtctttgtgctgtccaggttaatgccacgtctcaaatttt

aagaaaaaataaaatcaaaacataacatataataattaaaatgattaaaaatcaat

gaattattataatttgtatgattttttaaatctat**gcaataacagcgaaa**tgtaca

actttactatcaaatctaaacttcaaaaaaacccaaaaacaacattttaatatata

tttttacatttggttacaagtttaacac**ttgctt**taagatttgtaatggc**tagatt**

gaaaaca**g**ttaaaagtattttcgtaaatatttttctctttctggataatgggctat

ttcaacccacagcagtgcaacatctgtcagtacttctggtgcctttattttatggg

ggcagctgtcagatgtgcgattaaaaaaagtggagtttcatcatg

Figure S2: DNA sequence of the *csgDEFG* regulatory region. Potential SoxS binding sites are in green and *csgDEFG* promoter elements are underlined.

Figure S3

pAMNF pAMNF-*soxS*

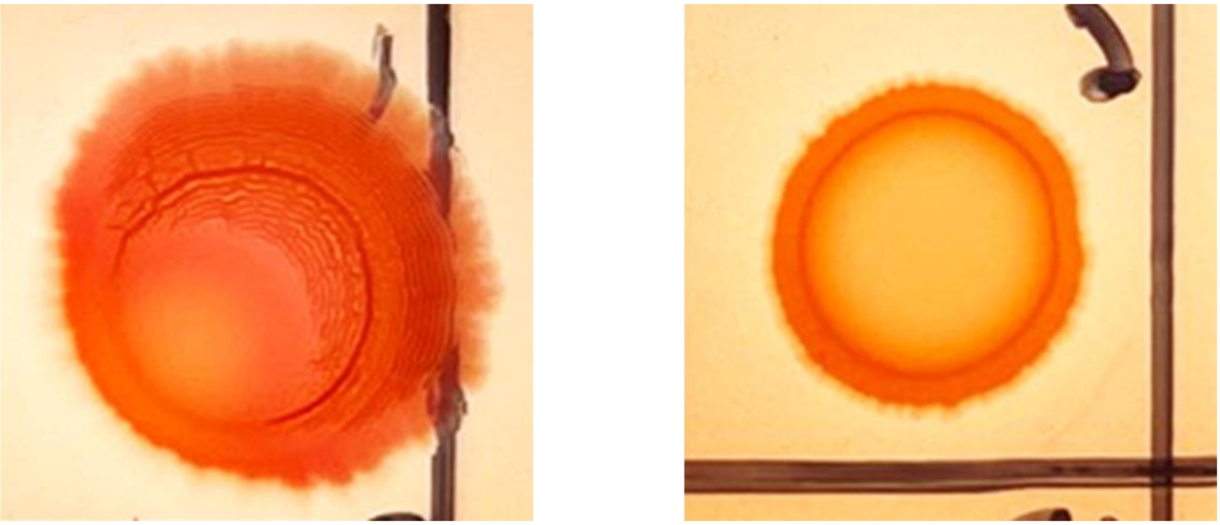

Figure S3: Macrocolonies formed by the indicated strains on agar plates containing Congo red dye.

Table S1: Strains, plasmids, and oligonucleotides

Name	Description	Source
	Bacterial strains	
Salmonella enterica subsp. *enterica* serovar Typhimurium SL1344	Histidine auxotroph of parental strain ST4/74	(1)
	Plasmids	
pAMNF	A pJ201 derivative encoding an N-terminal 3x FLAG tag upstream of HindIII and KpnI restriction sites. Contains an Ori_pUC origin and a kanamycin resistance cassette.	(2)
pAMCF	Derivative of pAMNF but encodes C-terminal 3x FLAG	(2)
pAMNM	Derivative of pAMNF but encodes N-terminal 8x Myc	(2)
pAMCM	Derivative of pAMNF but encodes C-terminal 8x Myc	(2)
pRW50T	LacZ reporter plasmid that encodes tetracycline resistance	(3)
pET28a	Protein expression vector with T7*lac* promoter	Novagen
	Oligonucleotides for cloning genes in pET28a (5'-3')	
marA-F	gtaggacatatgTCCAGACGCAACACTGACGC	This work
marA-R	gccagtggatccCTAGTAGTTGCCATCCTTCAGCG	This work
soxS-F	gtaggacatatgTCGCATCAGCAGATAAATTCAGACCC	This work
soxS-R	gccagtggatccCTACAGGCGGTGACGGTAATCGCT	This work
rob-F	gtaggacatatgGATCAGGCTGGCATAATTCGCG	This work
rob-R	gccagtggatccTTAACGGCGAATCGGGATCAGAAATT	This work
ramA-F	gtaggacatatgACCATTTCCGCTCAGGTTATCG	This work
ramA-R	gccagtggatccTCAATGCGTACGGCCATGCTTTTCTTTA	This work
	Oligonucleotides for cloning genes in pAM derivatives (5'-3')	
MarA N-terminal F	actgcaggtaccATGTCCAGACGCAACACTGACGC	This work
MarA N-terminal R	tgcagtaagcttCTAGTAGTTGCCATGGTTCAGCGGC	This work
soxS N-terminal F	actgcaggtaccATGTCGCATCAGCAGATAATTCAGACCC	This work
soxS N-terminal R	tgcagtaagcttCTACAGGCGGTGACGGTAATCGC	This work
Rob N-terminal F	actgcaggtaccATGGATCAGGCTGGCATAATTCGCG	This work
Rob N-terminal R	tgcagtaagcttTTAACGGCGAATCGGGATCAGAAATTCGC	This work
RamA N-terminal F	actgcaggtaccATGACCATTTCCGCTCAGGTTATCG	This work
RamA N-terminal R	tgcagtaagcttTCAATGCGTACGGCCATGCTTTTCTTTACG	This work

Oligonucleotides for cloning promoter fragments in pRW50T (5'-3')

csgD-F	ggctgcgaattcGCTGTCACCCTGGACCTGGTCG	This work
csgD-R	cgcccgaagcttCATGATGAAACTCCACTTTTTTTAATCGC	This work
M1	GTATGATTTTTTAAATCTATGCAATCCCATAGCCCTGTACAA CTTTACTATCAAATC	This work
M2	GGGGATGTTCTTATGCTTCCCATGTGGGGCAATACGCACACC ACTAGCCCCACTTCGTTTTTTTTGTCTTTGTGCTGTCCAGG	This work

References

1. Hoiseth,S.K. and Stocker,B.A.D. (1981) Aromatic-dependent Salmonella typhimurium are non-virulent and effective as live vaccines. *Nature*, **291**, 238–239.
2. Sharma,P., Haycocks,J.R.J., Middlemiss,A.D., Kettles,R.A., Sellars,L.E., Ricci,V., Piddock,L.J. V and Grainger,D.C. (2017) The multiple antibiotic resistance operon of enteric bacteria controls DNA repair and outer membrane integrity. *Nat. Commun.*, **8**, 1444.
3. Manneh-Roussel,J., Haycocks,J.R.J., Magán,A., Perez-Soto,N., Voelz,K., Camilli,A., Krachler,A.M. and Grainger,D.C. (2018) Camp receptor protein controls Vibrio cholerae gene expression in response to host colonization. *MBio*, **9**.
